# Complexity of FGFR signalling in metastatic urothelial cancer

**DOI:** 10.1186/s13045-015-0221-6

**Published:** 2015-10-24

**Authors:** Alejo Rodriguez-Vida, Matilde Saggese, Simon Hughes, Sarah Rudman, Simon Chowdhury, Neil R. Smith, Peter Lawrence, Claire Rooney, Brian Dougherty, Donal Landers, Elaine Kilgour, Hendrik-Tobias Arkenau

**Affiliations:** Guy’s and St Thomas’ NHS Foundation Trust, London, SE1 9RT UK; Sarah Cannon Research Institute UK, London, W1G 6AD UK; University College London Hospital, London, NW1 2BU UK; Oncology iMED, AstraZeneca, Alderley Park, Macclesfield, Cheshire SK10 4TG UK; Oncology iMED, AstraZeneca, Gatehouse Park, Boston, USA; Hospital del Mar, Barcelona, 08003 Spain

**Keywords:** Urothelial cancer, FGFR, AZD4547, Biomarker

## Abstract

**Background:**

Urothelial cancers (UC) are the fourth most common tumours worldwide after prostate (or breast), lung and colorectal cancer. Despite recent improvements in their management, UC remain an aggressive disease associated with a poor outcome. Following disease progression on first-line platinum-based chemotherapy, very few effective treatment options are available and none of them have shown significant improvement in overall survival. Alterations of the fibroblast growth factor receptor (FGFR) pathway including amplification, mutations and overexpression are common in UC. Pre-clinical data suggest that the presence of such dysregulations may confer sensitivity to FGFR inhibitors.

**Materials and methods:**

We present here the case of a patient with a metastatic UC of the renal pelvis with lymph node metastases treated with the selective FGFR inhibitor AZD4547.

**Results:**

To date, the patient has been on a study drug for 32 months with acceptable tolerance and maintained radiological partial response as per RECIST 1.1 criteria. Exploratory biomarker analysis showed FGFR3, FGFR1, FGF-ligand and fibroblast growth factor receptor substrate 2 (FRS2) expression in the patient’s tumour, together with the presence of a germ-line mutation in the FGFR3 extracellular binding domain. This is not a known hotspot mutation, and the functional significance remains unclear.

**Conclusions:**

The FGFR inhibitor AZD4547 exhibits antitumour activity in a metastatic urothelial cancer displaying FGFR1, FGFR3, FGF-ligand and FRS2 expression. This lends support to the further exploration of FGFR inhibitors in urothelial cancer. Further studies are required to determinate the most effective way to select those patients most likely to respond.

## Background

Urothelial cancers (UC) are the fourth most common tumours worldwide after prostate (or breast), lung and colorectal cancer [1] and originate along the transitional epithelium from the renal pelvis to the ureter, bladder and proximal two thirds of the urethra. Whilst bladder tumours account for 90–95 % of UC, upper tract urothelial cancers (UTUC) involving the renal pelvis and ureter are rare, representing only 5–10 % of all UC. Contrary to the improvement seen in overall survival (OS) in the last years in many other cancers, UC remains an aggressive disease associated with poor outcomes. Following radical surgical resection, the 10-year OS is 20–60 % for bladder cancer and 25 % for locally advanced UTUC [2, 3]. Similarly, following disease progression on first-line platinum-based chemotherapy combinations, very few effective treatment options are currently registered for metastatic UC, and none of them have shown significant improvement in OS. There is therefore an important unmet need for effective anticancer treatment in advanced UC. However, recently impressive clinical responses and progression-free survival benefit have been reported for both pembrolizumab and atezolizumab in UC patients who have failed first-line chemotherapy [4, 5]. These agents target the PD1/PDL1 T-cell checkpoint, and it is likely that immunotherapies will represent a significant advance in the treatment of metastatic UC patients. However, not all patients respond to these therapies, and work remains to be done to determine the molecularly defined disease segments which are sensitive to immunotherapies.

In the last decade, numerous targeted therapies have been approved for the treatment of metastatic solid cancer such as breast, colon, melanoma or kidney cancers among others. However, despite the existence of various potential targetable molecular alterations such as in the epidermal growth factor receptor (EGFR), the mammalian target of rapamycin (mTOR) or the aurora A kinase pathways, no targeted agents have proven to be of clinical benefit for patients with UC. The fibroblast growth factor receptor (FGFR) pathway has also been extensively studied in UC. The FGF/FGFR signalling axis comprises of 18 ligands which bind to four highly conserved trans-membrane tyrosine-kinase receptors (FGFR1, 2, 3 and 4). Fibroblast growth factors (FGF) signalling through their cognate receptors play an important role in normal organ, vascular and skeletal development and in the homeostatic control of phosphate and bile acids. Genetic alterations of the FGFR genes including amplification, translocation and mutations promote cell proliferation, cell migration, anti-apoptosis and angiogenesis and have been described in a range of tumour types including urothelial cancers [6] (Table 1). Amplifications of the FGFR1 gene have been found in 9–10 %, FGFR2 gene in 0.8 % and FGFR3 gene in 3–5 % of UC cases [6–8]. FGFR3 has been shown to harbour activating mutations in 38–66 % of non-invasive UC and in 15–20 % of invasive UC, and a low prevalence of FGFR-gene fusions has been reported in UC [6, 9]. In addition, elevated expression of both FGFR1 and FGFR3, independent of FGFR gene amplification and mutation events, is reported to occur in a significant proportion of UC [6, 9]. In pre-clinical models, the presence of FGFR mutations, fusions and overexpression confers sensitivity to FGFR inhibitors [10, 11].

**Table 1:** Genomic abnormalities of the FGFR pathway in cancer

Gene		Cancer	Prevalence (%)
FGFR1	Amplification	Hormone receptor positive breast cancer	10–15 [20]
		Squamous NSCLC	10–20 [21]
		Urothelial cancer	9–10 [7]
		Squamous cell carcinoma of the head and neck	10–17 [22]
		Oesophageal squamous cell carcinoma	9 [23]
		Osteosarcoma	5 [11]
FGFR2	Amplification	Gastric cancer	5–10 [24]
		Triple negative breast cancer	4 [25]
	Mutation	Squamous NSCLC	3–5 [26]
		Endometrial cancers	12 [27]
FGFR3	Amplification	Urothelial cancer	3–5 [7]
	Translocation	Multiple myeloma	20 [28]
		Glioblastoma	3–7 [29]
	Mutation	Non-invasive urothelial cancer	38–66 [6, 9]
		Invasive urothelial cancer	15–20 [6–8]

*FGFR* fibroblast growth factor receptor, *NSCLC* non-small-cell lung cancer

We present a case of a patient with a metastatic UC and expression of the FGFR signalling pathway treated in a phase 1 trial with the FGFR inhibitor AZD4547. This patient was recruited into a phase 1 expansion arm study in advanced cancer patients with solid tumours harbouring either an FGFR1 or FGFR2 gene amplification as defined by centralised fluorescence in situ hybridisation (FISH) screening. Preliminary reports of the results from this phase 1 study have been presented [12, 13], and a full manuscript is in preparation. Twenty-one patients were recruited into the Study 1C1 expansion arm, including three UC patients, two of whom experienced disease stabilisation (on-drug for 171 days and 32 months). The patient reported here experienced the more durable disease stabilisation.

## Case presentation

A 47-year-old man presented with painless haematuria. He was a current smoker but had no relevant comorbidities. A flexible cystoscopy demonstrated a neoplastic lesion in the left ureteric orifice. Biopsy revealed a poorly differentiated transitional cell UC. A chest and abdomen computerised tomography (CT) scan showed enlarged para-aortic lymph nodes and a 3-cm mass in the left renal pelvis. He underwent a radical left nephroureterectomy and lymphadenectomy. Histopathology assessment reported a grade 3 multifocal papillary urothelial carcinoma of the renal pelvis and one metastatic left iliac lymph node. The final pathological stage was pT3pN1. He completed four cycles of adjuvant chemotherapy with cisplatin and gemcitabine with no major toxicities. Nine months later, a CT scan demonstrated disease recurrence with prominent new metastatic mediastinal, retroperitoneal and pelvic lymph nodes.

**Fig. 1:**
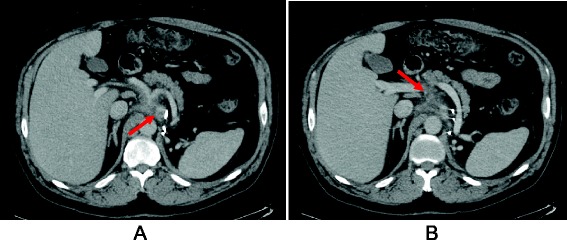
Baseline CT scan. Baseline CT scan showing the two selected target lesions as per RECIST 1.1 criteria: a left coeliac lymph node measuring 1.6 cm in the short axis (**a**) and a soft tissue mass next to the superior mesentery artery measuring 5 cm (**b**)

Given his good performance status, he was referred for consideration of a phase 1 trial. He was considered for the expansion phase of an open-label phase 1 trial testing the antitumour activity of the FGFR inhibitor AZD4547 in patients with FGFR1- and/or FGFR2-gene-amplified advanced solid malignancies. He underwent pre-screening testing of his FGFR status using FISH in an archival tumour tissue block containing a metastatic iliac node. According to the trial protocol, FGFR was considered to be amplified if the ratio between the FGFR gene copy number and the centromere probe count (FGFR/CEP10) was ≥2.0 across 50–100 tumour cell nuclei counted or if the percentage of tumour cells containing large FGFR clusters was ≥10 %. Molecular testing revealed an amplification of the FGFR1 gene as per the FGFR/CEP10 cluster definition. The complete molecular findings are summarised in Table 2. A baseline CT scan was performed, and two target lesions were selected as per RECIST 1.1: a left coeliac lymph node measuring 1.6 cm in the short axis and a soft tissue mass next to the superior mesentery artery measuring 5 cm (Fig. 1). After confirmation of his eligibility, he was started on the study drug. According to the dose-escalation phase of the study, AZD4547 was administered orally at a dosage of 80 mg twice daily, every day, in 21-day cycles.

**Table 2:** Patient molecular screening showing complex dysregulation of the FGFR signalling pathway. FGFR1 FISH was performed by central screening laboratory (Quintiles). FGFR1 and FGFR3 protein levels were assessed by IHC and FGFR1, FGFR3 and FGF-ligand expression assessed by NanoString. Gene variants and copy number gains were determined by next-generation sequencing analysis at Foundation Medicine

FGFR protein H-score	FGFR status (FISH)	FGF pathway RNA expression (NanoString)	Variants detected in tumour	Copy number gain (copy number, exons)
FGFR1: cytosol 150/membrane 0	No FGFR1 amplification (FGFR/CEP10 ratio 1.76)	High FGFR1 mRNA expression	FGFR3 (S236N)	MDM2 amplification (16, exons 11 of 11)
FGFR3: cytosol 10/membrane 1		High FGFR3 mRNA expression	ARID1A N399fs*218	MYC amplification (7, exons 5 of 5)
		High FGF7-ligand mRNA expression	FLT3 A291fs*6	TBX3 amplification (7, exons 8 of 8)
		High FRS2 mRNA expression	CHEK2 T367fs*15	
			BRCA2 Q1073R	
			FANCD2 Q1405L	

*FGFR* fibroblast growth factor receptor, *FISH* fluorescence in situ hybridisation, *IHC* immunohistochemistry

**Fig. 2:**
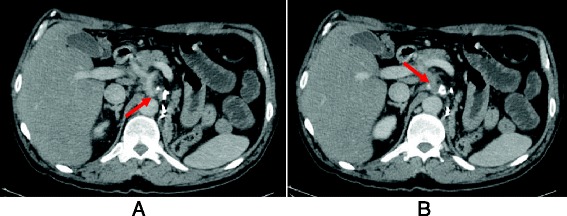
Assessment of response CT scan. CT scan performed at month 24 showing partial response to treatment as per RECIST 1.1 criteria: left coeliac lymph node measuring 1.0 cm in the short axis (**a**) and soft tissue mass next to the superior mesentery artery measuring 3.2 cm (**b**)

To date, the patient has been on study drug for 32 months with acceptable tolerance. The main adverse events encountered have been Common Terminology Criteria for Adverse Events (CTCAE) grade 1 hyporexia, grade 1 xerostomia and grade 2 nail toxicity which have all been successfully managed with supportive medications. No dose reductions or dose interruptions have been necessary. From the first trial CT scan, both target lesions have shown a progressive reduction in size. The best response to treatment was achieved at month 24, when the target lesions reduced in size to 1.0 and 3.2 cm for the left coeliac lymph node and the mesenteric soft tissue mass, respectively, with an overall 36.4 % reduction in tumour burden (Fig. 2).

**Fig. 3:**
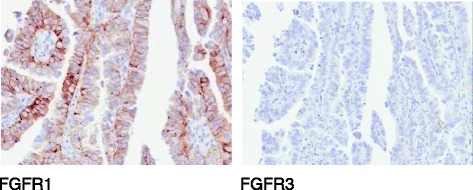
Example images of immunohistochemistry analysis of FGFR1 and FGFR3 in the tumour section

Two urine samples (one 12 h and one 24 h) were collected from the patient whilst on treatment with AZD4547. Renal excretion of AZD4547 in these samples was 2.53 and 2.33 %, respectively. The range of the fraction of dose excreted unchanged over the dose interval in part A of the phase 1 dose-escalation study was 1.7 to 8.4 % (overall mean 4.05 %); therefore, the fraction of AZD4547 renally excreted unchanged in this patient was minor and in keeping with these in the dose-escalation, making it unlikely that high intravesical concentrations of the drug accounted for the observed clinical benefit. Exploratory molecular analysis of the patient’s archival tumour sample included FGFR1 and FGFR3 protein expression by immunohistochemistry (IHC) and next-generation sequencing (NGS) analysis at Foundation Medicine. NanoString analysis of gene expression levels was performed on tumour samples from a total of 81 patients (15 patients dosed with AZD4547 plus an additional 66 patients pre-screened for the study). Exploratory biomarker results are summarised in Table 2. Analysis revealed expression of FGFR1 and FGFR3 at both the protein (Fig. 3) and ribonucleic acid (RNA) level. Expression of multiple FGF ligands was detected by NanoString with a particularly high expression of FGF7. Analysis by NanoString showed that compared to other samples in the 81 tumour tissue cohort FGFR3IIIb and FGF7 expression levels were at the 89th and 95th percentile, respectively.

**Fig. 4:**
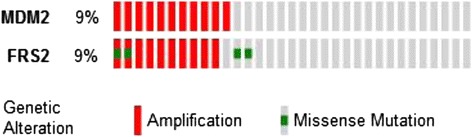
The Cancer Genome Atlas (TCGA) analysis. Analysis of The Cancer Genome Atlas (TCGA) data set showing 10 out of 11 MDM2-amplified urothelial cancers are also FRS2-amplified

**Fig. 5:**
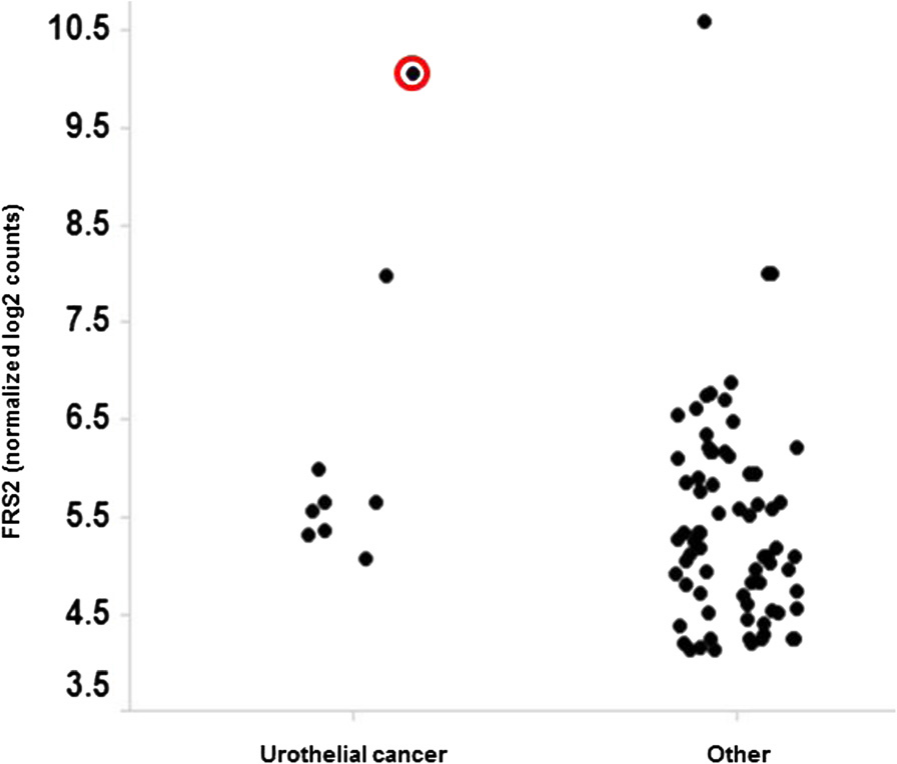
NanoString analysis of FRS2 expression. NanoString analysis of FRS2 expression in archival tumour samples from patients screened for the phase 1 study. Our case report patient is highlighted by a *red circle*

The gene mutation and copy number variants detected by NGS analysis are summarised in Table 2. Interestingly, this analysis failed to confirm the presence of FGFR1 gene amplification and detected the presence of an FGFR3 mutation (S236N). Subsequent analysis of DNA extracted from the patient’s blood sample showed this to be a germ-line FGFR3 mutation. In contrast to the known oncogenic FGFR3 mutations S249C, R248C and Y373C, expression of the S236N FGFR3 in MCF10 cells failed to induce anchorage-independent colony formation; hence, the functional significance of this mutation is unclear (data not shown). High-level amplification of murine double minute-2 (MDM2) was also detected. The fibroblast growth factor receptor substrate 2 (FRS2) gene lies close to MDM2 on chromosome 12q13-15, and these genes are often co-amplified. Analysis of The Cancer Genome Atlas (TCGA) data set shows that in UC, 10 out of 11 MDM2-amplified tumours are also FRS2-amplified [7] (Fig. 4) and NanoString analysis confirmed high FRS2 expression in our patient’s tumour sample (Fig. 5).

During the course of the clinical trial, the FISH scoring system of FGFR amplification was changed and the cluster definition of amplification was eliminated. Our patient’s FGFR status was reassessed using the new scoring system and showed a FGFR/CEP10 ratio lower than 2.0. Consequently, our patient was no longer judged to have a FGFR1 gene amplification. This is consistent with the fact that NGS analysis failed to detect an FGFR1 amplification. However, in view of the patient’s ongoing clinical benefit and tolerance of the study drug, and after discussing this issue with the patient, it was decided to continue treatment. Study treatment was ultimately stopped after 32 months on the drug due to disease progression in the existing lymph node disease with no new lesions elsewhere.

## Discussion

This report demonstrates for the first time that the FGFR inhibitor AZD4547 exhibits antitumour activity in a patient with metastatic UC. Moreover, a duration of response of 32 months is exceptional in the context of a metastatic UC in the first-line therapy setting. Exploratory biomarker analysis of an archival tumour sample showed clear evidence of high FGFR, FGF-ligand and FRS2 expression. FRS2 is an adaptor protein which lies downstream of the FGFR, mediating the activation of MEK and other signalling pathways, and FRS2 amplification is associated with sensitivity to FGFR inhibition [10]. AZD4547 is a potent selective inhibitor of the tyrosine-kinase activity of FGFR1, 2 and 3. FGF/FGFR signalling is known to be crucial in neoplastic development. FGFR is an increasingly studied oncogene with the potential to be involved in the proliferation of a significant proportion of tumours (Table 1).

In view of the pre-clinical evidence indicating oncogenic addiction in FGFR dysregulated xenografts, clinical development of FGFR inhibitors is now being focused in FGFR aberrant tumours. However, the preliminary results of phase 1 trials using selective FGFR inhibitors such as AZD4547, JnJ42756493 or BGJ398 have so far shown limited clinical benefit in FGFR aberrant tumours selected only on the basis of FGFR1 or 2 amplification (Table 3) with monotherapy response rates in the range of 5 to 25 % [13–16]. Moreover, pre-clinical studies in NSCLC have suggested that FGFR1 mRNA and protein expression might be better predictive biomarkers of response to FGFR TKI than gene copy number [17]. This case report illustrates the complexity of biomarker identification and drug development in oncology [18]. Further biomarker research is therefore needed in order to understand the best approach to patient selection in tumour types in which FGFR gene amplifications occur.

**Table 3:** Ongoing clinical trials with selective FGFR inhibitors

Agent	Phase	Clinicaltrials.gov	Description	Response rate	Disease stabilisation
AZD4547	Phase I trial	NCT00979134	FGFR1- and/or FGFR2-gene-amplified solid cancer (C1 cohort) [13]	1/20 (5 %)	9/20 (45 %)
			FGFR1-amplified squamous NSCLC (C2 cohort) [15]	1/15 (6.6 %)	5/15 (33.3 %)
			FGFR2-amplified gastric cancer (C3 cohort) [14]	1/13 (7.6 %)	4/13 (30.7 %)
AZD4547	Phase I/II trial	NCT01824901	FGFR1-amplified squamous NSCLC, randomised to docetaxel with or without AZD4547	NA	NA
AZD4547	Phase II trial	NCT01457846	Gastric or lower-oesophageal cancer, FGFR2 polysomy or amplification, randomised to AZD4547 or paclitaxel [30]	NA	NA
AZD4547	Phase I/II trial	NCT01202591	Oestrogen receptor positive and FGFR1-amplified BC, randomised to AZD4547 plus fulvestrant or fulvestrant alone	NA	NA
AZD4547	Phase I/II trial	NCT01791985	Oestrogen receptor positive BC, FGFR1-amplified or not, randomised to AZD4547 plus anastrozole or letrozole versus exemestane alone	NA	NA
AZD4547	Phase II study	NCT01795768	FGFR1- or FGFR2-amplified HER2-negative BC, NSCLC and gastroesophageal cancer [31]	3/9 (33 % in GC) 1/8 (12.5 % in BC)	NA
AZD4547	Phase II/III trial	NCT02154490	Squamous NSCLC, randomised to GDC-0032, rilotumumab, erlotinib, MEDI4736, palbociclib, AZD4547 or docetaxel depending on screening genomic analysis	NA	NA
AZD4547	Phase II trial	NCT02117167	Squamous NSCLC, randomised to AZD2014, AZD4547, AZD5363, AZD8931, selumetinib or vandetanib depending on screening genomic analysis	NA	NA
BGJ398	Phase I trial	NCT01004224	FGFR1- or FGFR2-amplified or FGFR3-mutated advanced solid tumours [19]	NA	NA
			FGFR1-amplified squamous NSCLC cohort [19]	4/26 (15.4 %)	9/26 (34.6 %)
BGJ398	Phase II trial	NCT01820364	Advanced melanoma, LGX818 followed by a rational combination with LGX818, MEK162, LEE011, BGJ398, BKM120 or INC280	NA	NA
BGJ398	Phase II trial	NCT02150967	Advanced cholangiocarcinoma, with FGFR2 gene fusions or other FGFR alterations	NA	NA
BGJ398	Phase II trial	NCT01975701	FGFR-amplified, translocated or mutated recurrent glioblastoma	NA	NA
BGJ398	Phase II trial	NCT02160041	FGFR aberrant solid tumours and/or hematologic malignancies	NA	NA
BGJ398	Phase I trial	NCT01928459	PIK3CA-mutated advanced solid tumours, without FGFR1–3 alterations, treated with BGJ398 with BYL719	NA	NA
BGJ398	Phase II trial	NCT02159066	Advanced melanoma, LGX818 plus MEK162 followed by a rational combination on progression with LEE011, BGJ398, BKM120 or INC280	NA	NA
LY2874455	Phase I trial	NCT01212107	Advanced cancer with FGFR aberrations during dose-expansion cohort	NA	NA
JNJ-42756493	Phase I trial	NCT01703481	Advanced cancer with FGFR1, 2 or 4 amplification (dose-expansion cohort) [16]	2/8 (25 %)	4/8 (50 %)

*FGFR* fibroblast growth factor receptor, *NSCLC* non-small-cell lung cancer, *BC* breast cancer, *GC* gastroesophageal cancer, *PIK3C* phosphatidylinositol-4,5-bisphosphate 3-kinase catalytic subunit alpha, *NA* not available

In phase 1 studies of FGFR inhibitors, an early efficacy signal has emerged in UC patients harbouring FGFR mutations or fusions [16, 19]. Here, we report on a urothelial cancer patient with FGFR1, FGFR3, FGF-ligand and FRS2 expression who has derived durable clinical benefit from AZD4547 therapy. A germ-line mutation in the FGFR3 extracellular binding domain was also detected, but in contrast to known hotspot FGFR3 mutations, this was not oncogenic when transfected into cells; hence, the functional significance is uncertain. This patient case highlights that, in addition to patients harbouring FGFR3 hotspot mutations or fusions in their tumour, there is potential for additional UC patients with high expression of FGFR pathway components such as FGFR, ligand and FRS2 to gain benefit from FGFR inhibitor therapy. Together, FGFR3 mutations, fusions or overexpression and FRS2 gene amplification occur in >50 % of urothelial cancer patients [6, 9], and further work is required to determine the optimal patient selection criteria for defining the sensitive patient population. Recently, encouraging clinical data has emerged for immunotherapies such as pembrolizumab and atezolizumab in advanced UC patients, and it will be interesting to understand the efficacy of these molecules in patients with FGFR pathway aberrations and the potential for combination with FGFR inhibitors. To address this, a clinical trial is now planned to explore the efficacy of AZD4547, both in monotherapy and in combination with the anti-PDL1 antibody MEDI4736, in advanced UC patients with FGFR3 mutations or fusion-positive tumours, and a future opportunity might be to expand these studies to patients with elevated FGFR and/or ligand expression.

## Conclusions

In conclusion, our case report illustrates the molecular complexity of the FGFR pathway. The FGFR inhibitor AZD4547 exhibits antitumour activity in a metastatic urothelial cancer displaying FGFR1, FGFR3, FGF-ligand and FRS2 expression. This is important as it lends further support to the exploration of FGFR inhibitors in urothelial cancer. Further work is required to optimise the predictive biomarkers of response to FGFR inhibitors in order to better select patients to clinical trials and ultimately provide them with a greater probability of deriving clinical benefit.

## Consent for publication

Written informed consent was obtained from the patient for publication of this case report and any accompanying images. A copy of the written consent is available for review by the Editor-in-Chief of this journal. Approval for publication was also obtained from AstraZeneca.

## Abbreviations

BC: breast cancer
CT: computerised tomography
FGFR: fibroblast growth factor receptor
FISH: fluorescence in situ hybridisation
FRS2: fibroblast growth factor receptor substrate 2
MDM2: murine double minute-2
NGS: next-generation sequencing
NSCLC: non-small-cell lung cancer
OS: overall survival
RNA: ribonucleic acid
UC: urothelial cancer
